# Clinical and Pathological Report on the Pneumonia of Children as It Prevails among the Poor in London

**Published:** 1843-09

**Authors:** Charles West

**Affiliations:** Physician to the Royal Infirmary for Children, and to the Finsbury Dispensary


*Clinical and Pathological Report on the Pneumonia of Children as it prevails among the Poor in London.* By CHARLES WEST, M.D., Physician to the Royal Infirmary for Children, and to the Finsbury Dispensary.*

PART FIRST.

If any person were to estimate the importance of pneumonia in early life from the space allotted to it in English works on the diseases of children, he would doubtles conclude that a malady concerning which the most experienced thought it necessary to say so little can neither be grave in its character nor frequent in its occurrence. Our tables of mortality, however, show that pneumonia is the cause of a larger number of deaths in childhood than any other diseases, with the exception of the exanthemata. It appears from the Appendix to the Third Report of the Registrar-General, that during the year ending May 22, 1841, 22,429 persons died within the Metropolitan districts under the age of fifteen. Of these 3058, or 13.6 per cent. died of pneumonia; 2963, or 13.0 per cent. of convulsions; and 1215 or 5.4 per cent. of hydrocephalus. A very similar result is obtained by examining the returns from Manchester, Liverpool and Birmingham, which are contained in the same report. In the year 1839, 11,164 deaths took place in these three towns, of persons under the age of fifteen. Of these persons 1348 or 12 per cent. died of pneumonia, 1615 or 14.4 per cent. of convulsions, and 493 or 4.4 per cent. of hydrocephalus. The slight

---

* We give insertion, in our present number, to a longer paper than has ever before found its way into this department of the Journal. We do so because that we look upon the production as one of great merit; and we feel assured that those of our readers who shall give it an attentive perusal, will not require of us any apology for republishing it at length.—EDTS.

in this case of deaths from convulsions cannot be regarded as invalidating the statement made as to the extreme frequency of pneumonia; but depends doubtless on the difficulty of obtaining an accurate return of the causes of death in places where there is a large proportion of migratory population, such as the Irish in Liverpool. That this is the true explanation of the apparent excess of deaths from convulsions appears further on observing that in Birmingham, where no such disturbing cause exists, and where the registers may therefore be presumed to be kept with greater accuracy, the per centage of deaths under fifteen years of age from convulsions is only 7.0, and from hydrocephalus 4.0, but from pneumonia 16.0 per cent. Even though the above returns were not completely exact, they yet must be regarded as affording a close approximation to the truth, and as establishing the serious nature as well as the frequent occurrence of inflammation of the lungs in childhood. A very large number of cases of this disease came under my notice at the Finsbury Dispensary, in the years 1841 and 1842, and at the Children's Infirmary in 1839–42, and the results of what I believe that I then learned are embodied in the following observations. For the opportunity of investigating both this and many other diseases of infancy during many years previous to my appointment to the office of physician to the Children's Infirmary, I am indebted to the great kindness of my friend and predecessor Dr. Willis; and gladly acknowledge the extent of an obligation which I can never hope to repay.

It will probably occur to some on reading the following remarks, that a great discrepancy exists between many of the statements and opinions they contain, and those of French writers of deserved reputation. To account for this, however, it will not be necessary to impugn the accuracy of the observations of either party; since two causes may be assigned fully adequate for its explanation. These are the very tender age of many of the infants who came under the notice of our continental neighbours; and the extremely unfavorable hygienic conditions in which all children are placed, whether at the *Hospice des Enfans Trouvés*, or at the *Hôpital des Enfans Malades*. The investigatiens of Billard and Valleix were carried on at the Foundling Hospital, and consequently for the most part on children only a few days old; while no child labouring under pneumonia who came under my notice was less than a month old. It should too be borne in mind that the morbid condition of the lungs of new-born children, which depends on their imperfect expansion at birth,

(first described under the name of atelectasis pulmonum by Dr. E. Jörg,*) was unknown either to Billard or Valleix, and that even now the French are by no means familiar with it.† But the condition of those children who are admitted into Foundling Hospitals is precisely such as most frequently gives rise to this affection; and, as Hasse‡ has shown in his elaborate work on Morbid Anatomy, the symptoms noticed during life, and the appearances found after death in many of the cases described as pneumonia by French writers, are exactly such as characterize atelectasis pulmonum.

Ample evidence exists of the extremely unfavorable conditions under which children are placed in the Children's or Foundling Hospital at Paris. One result of this circumstance is shown in the endemic prevalence of diseases in those institutions such as do not exist elsewhere; another in the frequent complication of almost all diseases with other secondary affections. Of these secondary affections gastro-enteritis and pneumonia are the most frequent. So often indeed is a condition of the lungs resembling that produced by inflammation observed among the inmates of the Foundling Hospital, that some have asserted pneumonia to be an invariable complication of the diseases of new-born infants.§ The researches too of M. Becquerel,‖ which were carried on at the *Hôpital des Enfans Malades*, where none of the patients are under two years of age, discovered traces of inflammation of the lungs in the bodies of 49 out of 133 children who had died of various affections. From these facts and some others of a like nature, M. Becquerel concludes that pneumonia supervenes rarely in perfectly healthy children; that it occurs most frequently in those who are exhausted by previous disease, or placed in unfavorable hygienic conditions; and that it comes on in the course of acute diseases of specific adynamic character. To this opinion French writers almost universally subscribe, and some have even denied the existence of idiopathic pneumonia in children between two and five years of

---

* In his Dissertatio de pulmonum vitio organico—Lips. 1832; and afterwards more fully in his work, Die Fötuslunge im gebornen Kinde.—Grimma, 1835.

† In proof of this, see a paper by M. A. Lhommeau, in Gaz. des Hôpitaux, Sept. 20, 1842, "Sur un état particulier du poumon chez un nouveau né," wherein he describes minutely a case evidently of Atelectasis; and notices its difference from pneumonic lung, but is quite at a loss as to its real nature.

‡ Specielle pathologische Anatomie. Band i.—Leipsig, 1841, s. 324–35.

§ See a paper by M. Savatier, in La Clinique for 1834, republished in Froriep's Notizen, Bd. xix No. xxi., also Valleix Clinique des Malad. des Enfans. Paris, 1838. Chap. ii.

‖ Archives Générales de Médecine, 1839, p. 437.

age.* MM. Rilliet and Barthez† likewise, though they do not agree to this proposition as absolutely true, yet admit that it is subject to but very few exceptions, since they met with only 3 instances out of 40 cases of pneumonia which occurred in children between two and five years of age, where the patients were previously in good health. Even between the ages of six and fifteen, idiopathic pneumonia according to their observations is rare, 6 only out of 20 children having been free from other disease at the time of its invasion.‡

It may suffice to have mentioned these facts without enlarging on the different features which disease must present among children who for the most part were not suffering from extreme poverty, and who were tended by their parents at their own homes, from those which it wears among the wretched inmates of the Foundling and Children's Hospital at Paris.

Morbid Anatomy. I will now proceed to detail the results of my own observations; prefacing them with a Tabular View of 37 *post-mortem* examinations of children who died of pneumonia. The appearances are arranged under the heads of Lobar, Lobular, or Vesicular Pneumonia, according as one or the other rorm of inflammation predominated.

[The tables here alluded to occupy nine pages, containing the details of the *post-mortem* examinations of the bodies of 37 children. From them we gather that 22 cases presented instances of *lobar* pneumonia; 9 males and 13 females. Two were of less than one year old; 9 between the ages of 1 and 2 years; 2 between 2 and 3; 3 between 3 and 4; 3 between 4 and 5; 1 between 5 and 6; 1 of 9; and one of 11. Thirteen of these cases (*lobar* pneumonia) were idopathic. The remaining 9 followed various diseases; one followed coryza in a phthisical child; one followed symptoms of gastro-enteritis; one supervened on phthisis; one supervened on measles complicated with diphtheritis; one complicated measles which

---

* Gerhard, in American Journal of Medical Sciences, August and November, 1834; and Rufz, Journal des Connaissances Médico-chirurgicales, 1835, p. 101.

† Maladies des Enfans, Aff. de Poitrine.—Paris. 1838, p. 76.

‡ Since this was written, MM. Rilliet and Barthez have published their Traite Clinique et Pratique des Maladies des Enfans, and the statements contained in it show the frequency of idiopathic pneumonia to be greater than they had formerly supposed. "Of 245 children attacked with pneumonia, 58 were previously in good health, 24 of these 58 children were under five years old, 34 had exceeded that age."

supervened on hooping-cough; one supervened on acute hydrocephalus in a phthisical child; two accompanied dropsy after scarlatina; and one supervened in course of acute rheumatism.

Eleven out of the 37 presented instances of *lobular* pneumonia; 5 males, and 6 females. Out of these, 5 were idopathic; 5 followed hooping-cough; and 1 accompanied measles which followed croup.

Four out of the 37 presented instances of *vesicular* pneumonia; 2 males, and 2 females. Three of these were idiopathic, and the other followed hooping-cough.

As the remaining details of the tables occupy so much space, and as suitable inferences are drawn from them by our author, we have deemed it best to omit them.—Edts.]

No one can be more sensible than I am of the many deficiencies in the above tables, and I can only plead in extenuation that almost all the *post-mortem* examinations were made without assistance, in the houses of the poor, whose prejudices and suspicions often rendered it impossibible to devote to such investigations more than a small portion of that time which they require. The facts, however, though incomplete, may be relied on, since in no case are they stated from recollection, but always from notes taken on the spot.

The first conclusion which may fairly be deduced from these data is, that the lobar pneumonia attacks children much more frequently, in comparison with the other forms of the diseases, than has been imagined by French writers; and that differences of age do not occasion such a liability to one form of inflammation of the lungs, and such an immunity from the other form as would appear from their statements. Of twenty-two *post-mortem* examinations of cases of lobar pneumonia, nineteen are of children under five years of age, and ten of children under two; while two of the *post-mortem* examinations of cases of lobular pneumonia are of children between the ages of six and seven.

A more striking difference between the two classes of cases appears in the fact that five of the cases of lobular pneumonia supervened on hooping-cough, and that in another instance the disease complicated an attack of measles which succeeded to croup. In the remaining five cases indeed, it is said to have been idiopathic, but even here the bronchi were found either greatly injected or containing a very abundant secretion in their cavities. These circumstances give considerable probability to the supposition of some Freneh writers, that lobular pneumonia occurs as the result of the extension of inflammation of the bronchi to the substance of the lung,—a theory which would explain many of the peculiarities that distinguish it from lobar pneumonia. Its greater prevalence during infancy and early childhood than subsequently, might perhaps be accounted for in accordance with this hypothesis by the extreme frequency of catarrh among young children, and by the fact that in the great majority of cases hooping-cough and measles occur before the commencement of the second dedtition.

The appearances produced by lobar pneumonia in the child do not require particular notice, since they differ in no important respect from those observed in the adult.

I once met with a condition of the lung, (which I believe to be very rare), closely resembling what some have described as chronic pneumonia. The subject of this observation had suffered from cough and difficult breathing for a month before she came under my care, and died ten days afterwards. On examining the body a small quantity of clear serum was found in the cavity of the left pleura, and a few easily broken adhesions existed between the left lung and the ribs. The right pleura contained from ℥ij. to ℥iij. of a turbid sero-purulent fluid, and the lung was invested with a thin layer of yellow lymph, by which it was in many parts connected with the ribs. The upper two thirds of the upper lobe of the left lung were slightly congested, the lower third was in a state of gray hepatization, with purulent depôts in many of the pulmonary vesicles, constituting the state described as vesicular pneumonia or vesicular bronchitis by different authors. The left lower lobe was in the first stage of pneumonia. The different lobes of the right lung were adherent to each other. The lower lobe was in the first, the middle in the third stage of pneumonia. The upper lobe was perfectly solid, of a light gray colour, resembling very much a piece of soap, smooth when cut, not soft, but easily broken. In its substance were patches of a red colour, like wine lees; soft and pultaceous to the touch, and breaking down into a kind of quagmire, in which no trace of pulmonary tissue was discernible. The lower edge of the lobe had generally this red appearance and pultaceous consistence, but little patches of it, some not bigger than a pea, were diffused through its substance in various parts. Neither the lungs nor any other organ in the body showed any trace of tubercle.

The condition to which the appearances in this case bear the closest resemblance is not that gray form of chronic pneumonia described by Andral, in which the lung retains its granular structure; but a state which has been described by Hasse in his Morbid Anatomy. That writer says,* that sometimes he has found "in children who have had symptoms of inflammation of the lungs, a light gray, nearly white, or yellow, induration of a whole lobe or of several lobules, which seems to affect the upper lobes more frequently that the lower." The appearance is quite different from that sometimes met with in children when a whole lobe is converted by tubercular deposit into a solid, cheesy, substance; and I am the less disposed to consider the change as any variety of tubercular degeneration, owing to the absence of tubercle from all other parts of the body.

There is a condition of the lungs, described under the name of *carnification* by MM. Rillet and Berthez, and regarded by them as of frequent occurrence. I have also met with it, though neither so frequently, nor involving so large an extent of pulmonary tissue as in the cases described by those gentlemen. I have not, however, included it in the table of *Post-Mortem* Appearances, since some of the examinations there recorded, were made before I had met with their work, and consequently before my attention had been especially directed to it. They describe portions of lung which have undergone this change as being depressed below the level of the surrounding tissue, of a violet colour, presenting when cut a smooth, red surface, yielding a bloody serum when squeezed, in which no air bubbles are contained, and in short resembling a portion of muscle. The comparison they make of lung in this condition, with a portion of fœtal lung is very exact; its appearance indeed is precisely such as is observed in infants who have died from Atelectasis Pulmonum. The advanced age of many of the patients in whom it was noticed by MM. Rilliet and Barthez, as well as by myself, forbids us to attribute it to that cause. They suggest that it may be a form of chronic pneumonia, but this supposition can hardly be considered tenable, if we bear in mind that in many of the instances in which it was observed the inflammatory process ran its course with great rapidity.

---

* Op. cit. p. 292.

Neither does it seem attributable to the pressure of fluid effused into the pleura, since in one only of MM. Rilliet and Barthez's eleven cases in which the lung was carnified did there exist any effusion into the sac of the pleura;* a statement which my own experience fully confirms. This condition usually presented itself to me affecting a cluster of two or three lobules in the substance of a lung, or more frequently fringing the lower edge of a lobe; the lower edge of the upper er middle lobe being its seat more frequently than the lower edge of the lower lobe. I would not have alluded to a condition on the real nature of which I can throw so little light, but from the hope that the attention of others more favourably circumstanced for the pursuit of morbid anatomy may thereby be directed to the subject.

Closely allied to lobar pneumonia is that œdematous condition of the lung, not unfrequently met with in children dying with chest affections in the course of dropsy after scarlet fever. In most cases where this state is found after death, the dyspnœa has either come on, or, at least, has been aggravated very suddenly, being attended with great distress, and exceedingly tumultuous action of the heart, and proving very rapidly fatal. A considerable quantity of serous fluid is generally found in each pleura, both lungs are universally congested, and gorged with bloody, frothy fluid, which exudes abundantly on cutting into them. This is evidently not the result of mere position, since the upper are quite as much affected as the lower lobes. The congestion appears to exist in the same degree everywhere, and though the texture of the lung may be less firm than natural, yet I have never found any portion in a state of actual hepatization.

Lobular Pneumonia. It has not been attempted in the table of morbid appearances to make that distinction between cases of simple and of generalized Lobular Pneumonia which has been drawn by MM. Rilliet and Barthez. It will be seen by a reference to the table, that in almost every instance, even if the inflamed lobules in one lung were distinct, the pneumonia had become generalixed in the other. Perhaps, too, MM. Rilliet and Barthex would have found this to be the case more frequently, if the children who came under their notice had been placed in conditions more favorable to their bearing

---

*These gentlemen base their observations in their recent work on forty-two instances of carnification, and they expresely state that their remarks do not refer to that form of the lesion which is the mechanical result of effusion into the pleura. (Op. cit. t. i. p. 74.)

up under the disease, and if time had thus been given for the lobular pneumonia to become generalized.

*First and second stages.* A lung affected with Lobular Pneumonia presents a mottled appearance, portions of a deep red colour being interspersed in the midst of others having a natural aspect. This condition closely resembles that of a lung which is the subject of Atelectasis, but there is one point of difference by which the two states may very generally be distinguished from each other. In each, the dark portions of the lung are depressed beneath the general level; but in Atelectasis this depression is real and owing to the dark portions never having been expanded by the entrance of air; in Lobular Pneumonia it is apparent only, being produced by the emphysematous distention of the surrounding tissue. A section of the lung presents an appearance similar to that of its surface, and shows even more clearly that the red portions are inflamed lobules, and the pale lobules those which have not been the seat of inflammatory action. It happens indeed comparatively seldom, that single lobules are found affected, the inflamed patches usually comprising four or five, which together form a mass of the size of a nut or an almond. These portions of inflamed tissue give to the lung an unequally hard feel, such as is described, though apparently without its cause being understood, in Dr. Watt's valuable monograph.* It is there said in the account of the *post-mortem* appearances found in one case, that "The lungs felt knotty, and uncommonly firm in some places; but on cutting into them no tubercles, such as are met with in phthisis, could be detected." If the patient lives for some time, the intervening substance usually becomes affected and the lobular is thus converted into lobar pneumonia. This change does not appear to take place by the gradual extension of disease from each inflamed lobule as from so many distinct centres, in which case one would expect to see a gradual shading off of the inflammation from the dark, highly-inflamed centre, to the paler, less inflamed periphery; but sooner or later the whole intervening pulmonary substance seems at once to become the seat of inflammatory action which runs its course as in ordinary lobar pneumonia. It happens, indeed, sometimes in cases where an entire lobe is affected with lobar pneumonia in the first stage, that some dark-red and perfectly solid patches are found which might

* Treatise on Chincough. Glasgow, 1813, 8vo., p. 131.

seem to indicate that the disease began a lobular pneumonia. But even here, there is none of that gradual deepening in intensity of the inflammatory appearances as we approach each solid lobule which must needs exist if lobular pneumonia became general by involving the different lobules in succession. In a few cases in which pneumonia, originally lobular, was becoming general when death took place, I have found the pulmonary tissue intervening between the inflamed lobules "drier than usual, not at all engorged, as in Laennec's first stage, and of a bright-red colour from intense arterial injection;" in that condition in short, regarded by Dr. Stokes* as constituting really the first stage of pneumonia.

*Third stage.* In the greater number of cases of lobular pneumonia death occurs before the inflamed lobules have passed into the stage of gray hepatization, or the lobular pneumonia becomes general, and the third stage consequently presents no peculiarity. To this, however, there are occasional exceptions, the inflamed lobules either becoming infiltrated with pus, and then presenting on a small scale the same appearance as is seen on a large scale in ordinary gray hepatization; or each lobule becomes the seat of a small, distinct abscess; with numbers of which the lung seems riddled. These pulmonary abscesses were met with twice; both times in cases where pneumonia had supervened on hooping-cough. In one instance, they were found in both lungs and coexisted with extensive tubercular deposit in those organs. It might indeed be objected to this case, that the supposed vomicæ were in reality softened tubercles, though it is my belief that no such error was committed, and that none of the tubercles had passed the crude stage. Be that, however, as it may, no such objection exists in No. 8, in which the vomicæ existed only in the lower lobes of each lung, while no tubercle was found in any organ of the body. These collections of matter which vary in size from that of a millet-seed to that of a pea, are found in the centre of the lung as well as near its surface. They sometimes communicate distinctly with a bronchial tube, but at other times no such communication can be clearly traced. They are irregularly circular, not lined by any smooth membrane, nor surrounded by a barrier of indurated lung such as is often seen around small collections of softened tubercle. They may be further distinguished from tubercle by the circumstance that they usually occupy the lower lobes

* Diseases of the Chest. Dublin 1837, p. 311.

only, and that they are found in cases where all other organs are free from tubercle. It is decidedly a rare condition. MM. Rilliet and Barthez met with it only twice in forty-three *post-mortem* examinations;* and I but twice in thirty-seven.

Vesicular Pneumonia. That state of the lungs known by the name of Vesicular Pneumonia or Vesicular Bronchitis first excited general attention from a description of it contained in a dissertation by M. Lanoix. The appearance, however, is described by Dr. Watt† who had observed it and referred it to its true cause. A lung, or a portion of lung which is the seat of this affection, presents an uneven surface; the inequality being produced by the presence of a number of small, circular, yellowish prominences, which bear a considerable resemblance to crude tubercles. They may, however, readily be distinguished from tubercle, for not only do they almost always occupy the lower margins of the different lobes, but on puncturing any one of them with the point of a scalpel, a drop of pus will exude, showing them to be small collections of matter. The cavity in which these purulent collections exist appears to be that of the extreme pulmonary vesicles, a fact which may be ascertained by tracing a minute bronchus to its termination in one of these little sacs.

This condition is a very frequent complication both of lobar and lobular pneumonia, when it fringes the inflamed lobes, especially at their lower margins. Occasionally, too, it involves the whole of the middle lobe of the right lung, but it will be seen by a reference to the table that it seldom constitutes the chief lesion. In each of the four cases also in which vesicular pneumonia was the most prominent morbid appearance, the lungs presented signs of the other forms of pneumonia in a more or less advanced stage; the only exception to this rule being in No. 1, in which puriform secretion was contained in the pulmonary vesicles at the upper part of the upper lobe of the right lung uncombined in that situation with any of the other forms of pneumonia.

Complications. *Affections of the bronchi.* The state of the bronchi, and the nature of their contents were noted, though not with all the minuteness desirable in twenty-five cases. The following general results will be found, however, very nearly accurate. Some degree of increased redness of the air-tubes exists in most cases of pneumonia: in lobar

---

* In their recent work, they give the proportions as 26 in 314 *post-mortem* examinations of lobular pneumonia, p. 67.

† Op. cit. p. 139.

pneumonia, however, this redness is rarely intense, and is very often met with only in those situations where the substance of the lung itself is inflamed. In lobular pneumonia, intense congestion of the air-tubes is much more frequently observed, and is especially remarkable in the lobular pneumonia, which supervenes on pertussis. The bronchi are oftener empty in lobar than in lobular pneumonia, though in the former it is common to find puriform secretion in the bronchi near to any part which has passed into the third stage of pneumonia. Sometimes, too, abundant mucous fluid is found in the air-tubes while their lining membrane is quite pale; the accumulation of fluid in these instances being attributable to the inability of the patient to expectorate. In lobular pneumonia secretion of some kind or other is usually contained in the bronchi. It is oftener mucous than puriform, sometimes scanty, occasionally very abundant, and in this case it is not unfrequently very tenacious, and of a consistence approaching to that of false membrane. In these instances the secretion is usually more membraniform in the larger bronchi, more fluid in those of smaller caliber, which are sometimes rendered impervious to air by its quantity. I have found this secretion nearly approaching the membranous form only in two cases, in both of which it was associated with lobular pneumonia. Many points connected with this affection are undetermined; and some French writers* lean to the opinion of its being an essential disease, a true croup of the bronchi, and regard the pneumonia with which it is associated as a frequent but not necessary complication. I do not feel myself competent to offer an opinion on the subject, but will merely observe, that in neither of the two cases above alluded to were the symptoms observed during life different from those of ordinary pneumonia.

*Dilatation of the bronchi* was observed in eleven instances, but it was possibly overlooked in some cases where it did not exist in a very marked degree. This dilatation was never irregular, as it is occasionally in the adult, when it gives rise to an appearance which has been likened by Laennec to that of some marine fuci. It always presented the tubular form: was limited, when slight, to the smaller bronchi, but when considerable it likewise involved those of larger size. It

---

* See the cases and remarks of Jurine in Royer Collard's Rapport sur le Croup- 2ieme ed.—Paris, 1836, p. 34; and p. 222, No. viii. of the Appendix. Also M. Fauvel's Reecherches sur la Bronchite Capillaire, &c.—Paris, 1840, 4to.

will be seen by reference to the tables, that it was in cases where pneumonia had supervened on hooping-cough, that this dilatation existed in the most marked degree. The same tables also show that the measure of dilatation of the air-tubes bore no proportion to the amount of fluid they contained; a fact which may further prove that some theory other than that of their mechanical distention must be adopted in order to explain this occurrence. The change effected by the inflammatory process in the vital contracility of the tube is probably, as Dr. Stokes suggests, the chief cause of this condition, to which in cases of hooping-cough is superadded the influence of the violent inspirations which occur during the paroxysms of the cough.

*Emphysema.* The extreme frequency of emphysema as a complication of infantile pneumonia renders some notice of it necessary. It was found occupying the upper part of each lung, and frequently fringing the lower edge of each upper lobe. It was usually most considerable in cases where severe bronchial symptoms had existed, but was also very extensive in some cases which had never been marked by violent cough, but in which the inflammation involved a very considerable extent of lung, and ran a very rapid course. Interlobular emphysema was observed in four cases, sometimes simply dissecting the different lobules from each other, at other times giving rise to a number of vesicles containing air which covered the surface of the upper lobe of each lung.

*Pleuritis.* In 12 of the 37 cases no traces of inflammation of the pleuræ were found; in 5 there ixisted old adhesions more or less extensive, and in 20 there were the marks of recent inflammation. In 12 of the 20 cases both pleuræ were affected; in 6 adhesions existed only on the right, and in 2 only on the left side. One of the cases of double pleurisy might be characterized as slight; in 5 others the disease, though slight on one side, was extensive on the other, and in the remaining 6 extensive pleurisy existed on both sides. In 17 cases adhesions between the costal and pulmonary pleura and the presence of more or less lymph on the surface of the lung were the only signs of pleurisy; but in 8 cases there was also effusion of a notable quantity of fluid. In 3 instances this effusion was serous; in the remaining 5 it consisted of a turbid, sero-purulent or purulent fluid.

The above-mentioned results which agree very closely with those obtained by MM. Rilliet and Barthez show how little foundation exists for the statement which has been made, that

"there does not appear to be much tendency to pleuritis in the young subject."* This error has most likely arisen from Dr. Maunsell applying to children of all ages, that which is true only of those who are very young. It appears probable indeed, from the researches of M. Ch. Baron,† that the liability to pleurisy is much greater among children above 2 years of age, than among those who are younger; since "of 3392 autopsies of children from 1 to 2 years old, pleurisy was found only in 205, and in 79 the pleura was otherwise unhealthy from some non-inflammatory cause; or, in other words, inflammation of the pleura was found only in 6 per cent., while in 181 autopsies of children from 2 to 15 years old, the pleura of 158, or 87 per cent. presented signs of inflammation."

*Pericarditis.* Of the three instances in which pericarditis coexisted with pneumonia, one was a case of rheumatic pericarditis; in the other two the pericardium was probably affected by the extension of the inflammation to it from the pleura. M. Ch. Baron‡ mentions two instances in which he believes that this occurred, and two years ago a little girl was under my care in whom signs of pneumonia had existed for some days, and a pleural friction sound had been heard for a short time, but had again disappeared when a to-and-fro sound became distinctly audible, accompanying the heart's action, and continued to be heard until the patient died. Unfortunately a *post-mortem* examination could not be obtained.

*Tubercle.* Tubercles were found in 10 cases either in the lungs or the bronchial glands, or in both. In 5 they coexisted in the two situations, in 1 they were found only in the lungs; in 4 only in the bronchial glands, and in 1 of these 4 a single gland was affected, and that had undergone the cretaceous change. Comparatively rare, however, as it is to meet with tubercle in cases of acute inflammation of the lungs, there is a kind of bastard pneumonia which is by no means unusual in infants whose lungs have undergone very extensive tubercular degeneration, such as is seldom met with after the first two years of life. It comes on insidiously in infants at the breast, or in children in whom the process of dentition is not completed; its symptoms, consisting in some exacerbation of the previously existing fever and increase of the cough and dyspnœa, attract but little attention, and serious alarm is not

* Maunsell and Evanson on the Diseases of Children 2d edition, p. 309.
† De la Pleurésie dans l'Enfance. Paris, 1841, 4to, p. 52, Note 3.
‡ Ib. p. 49.

excited until it is seen how little they are amenable to any treatment. Its course is generally slow, occupying a fortnight or three weeks, but instances do occur in which the fatal termination takes place much more speedily. A lung which has been the subject of this morbid process presents a singular appearance; parts of it being of a solid texture, and yellowish white colour from tubercular deposits, interspersed with patches of a deep red hue, which are the lobules that were not involved in the tubercular degeneration, but which have become the seat of inflammation.

### PART SECOND.

Causes of Pneumonia.—I have now noticed all the chief points in the morbid anatomy of pneumonia which my own observation could serve to elucidate, and pass next to the examination of those circumstances which favour the development of the disease.

*Influence of season.* The season of the year has always been regarded as exercising considerable influence on the development of pneumonia, which has been generally supposed to be most frequent in the later winter and early spring months. The proportion borne by cases of pneumonia to all the cases, 2450 in number, that came under my notice at the Infirmary for Children, in 1841 and 1842, toking the mean of the two years, is shown to have been, during each three months, as follows:

| | |
|---|---|
| In 1st three months . . . . . | 5.1 per cent. |
| 2d ,, . . . . . . . . | 2.5 ,, |
| 3d ,, . . . . . . . . | 3.8 ,, |
| 4th ,, . . . . . . . . | 5.8 ,, |

Although this table does not quite accord with the generally received opinion as to the season when pneumonia is most prevalent, yet it tallies exactly with the results presented in the Third Report of the Registrar-general. It appears from that document, that the greatest mortality from pneumonia among persons under the age of fifteen years, takes, place in the month of December; and on a comparison of the different quarters of the year, it will be seen that the deaths from pneumonia are to the deaths from all causes, of persons under fifteen, in the following proportions:

| | |
|---|---|
| In 1st three months . . . . . | 15.3 per cent. |
| 2d ,, . . . . . . . . | 11.4 ,, |
| 3d ,, . . . . . . . . | 8.9 ,, |
| 4th ,, . . . . . . . . | 18.7 ,, |

*Age.* The age of the subject appears to exercise a very considerable influence in predisposing to pneumonia. During the years 1841 and 1842, 118 cases of idiopathic pneumonia came under my notice. The following table represents the ages at which these cases occurred, and the proportion per cent. they bore to the total number of cases of all diseases at corresponding ages.

From this table it appears that during the first five years of life, the cases of pneumonia bore the proportion of 10.3 per cent. to the total cases, while during the succeeding five years they were in the proportion of only 1.3 per cent. of the total cases. It will further be remarked, that during the first two years of life the proportion is as high as 17.5 per cent., and that the period when pneumonia is most prevalent coincides exactly with that during which the process of dentition is going on most actively; namely, from the sixth to the eighteenth month.

| Age. | Male. | Female. | Total. | Proportion to all Cases |
|---|---|---|---|---|
| Under one month . . ... | 0 | 0 | 0 | .0 per cent. |
| Between 1 and 2 months ... | 1 | 0 | 1 | 2.7 ,, |
| 2 3 ... | 3 | 0 | 3 | 6.5 ,, |
| 3 6 ... | 3 | 1 | 4 | 3.1 ,, |
| 6 12 ... | 19 | 6 | 25 | 8.7 ,, |
| 12 18 ... | 9 | 17 | 26 | 8.6 ,, |
| 18 2 yrs. ... | 5 | 6 | 11 | 5.5 ,, |
| 2 yrs. 3 ... | 11 | 10 | 21 | 7.8 ,, |
| 3 4 ... | 5 | 7 | 12 | 4.7 ,, |
| 4 5 ... | 3 | 4 | 7 | 3.9 ,, |
| 5 6 ... | 3 | 1 | 4 | 2.5 ,, |
| 6 7 ... | 1 | 0 | 1 | .8 ,, |
| 7 8 ... | 1 | 1 | 2 | 1.9 ,, |
| 8 9 ... | 0 | 0 | 0 | .0 ,, |
| 9 10 ... | 0 | 1 | 1 | 1.6 ,, |
| | 65 | 53 | 118 | |

A substantiation of the above statement is furnished by the Third Report of the Registrar-general. It appears from tables which that report contains, that of 1553 deaths from pneumonia which occurred in Liverpool, Birmingham, and Manchester, 1348, or 86.7 per cent. were of persons under fifteen. Of these 1348,—1093, or 81 per cent. were of children under the age of two; and 1231, or 91.3 per cent. of children under three years old.

The influence of age on the production of secondary pneumonia is a point on which I have not such facts as could be represented in the numerical form, but my decided impression is, that it is nearly the same in secondary as in idiopathic pneumonia, and the observations of MM. Rilliet and Barthez are favourable to this opinion.

*Sex.* Of the 118 cases which form the subject of the foregoing table. 65 occurred among boys, 53 among girls; and the bills of mortality for Manchester, Liverpool, and Birmingham show a similar excess of males among the deaths from pneumonia under puberty. This excess is somewhat greater than the actual excess of male births suffices to explain, although, if we calculate 105 male to 100 female births, it must be confessed that the influence of sex is at any rate very small.

*Previous health.* French practitioners have insisted so strongly on the importance of previous health as predisposing to pneumonia, that the subject must not here be passed over without notice. In 33 cases the previous health of the patients was ascertained, and found in 25 instances to have been good, in 7 delicate, and only in 1 decidedly bad. The conclusion, therefore, that a previously indifferent state of health predisposes to pneumonia, cannot be adopted with reference to children attacked by the idiopathic form of the disease in this country.

*Disease.* I regret that I have not data sufficient to enable me to state with perfect accuracy the proportion of cases of different diseases in the course of which pneumonia supervenes as a secondary affection. It is my belief, however, that (not reckoning catarrh, the influence of which will be presently considered), the chief diseases in the course of which inflammation of the lungs occurs may be ranged in the following order: Measles, Hooping-cough, Diarrhœa, and Remittent Fever. I can confirm the statement which has been made by others, as to the rarity of pneumonia in the course of scarlet fever, though the disease is by no means unusual in the course of the subsequent dropsy. In the few instances of small-pox which have come under my care, I have, in common with others, found inflammation of the lungs to be a most frequent and most fatal complication.

*Catarrh.* The authors of the most popular English work on the Diseases of Children say, that they "doubt much if pneumonia ever occurs in young children as a primary idiopathic affection;"* and express their opinion that it always comes on as secondary affection in the course of bronchitis. This assertion, coming with the weight which it necessarily

---

* Maunsell and Evanson, op. cit., p. 308.

receives from the deserved reputation of its authors, must, if erroneous, lead the practitioner into serious mistakes. That it is erroneous, I feel fully persuaded. In 50 cases of idiopathic pneumonia I have carefully noted the mode of attack, and find that in only 15 was it preceded by catarrhal symptoms. This fact is shown in the following table, as also is the other fact that the frequency of catarrh, as a prelude to pneumonia, is greatest while dentition is going on; that is to say, during the first two years of life.

| In Children of the following Ages. | Pneumonia was | | Total. |
|---|---|---|---|
| | preceded by Catarrh. | not preceded by Catarrh. | |
| Under 1 year, | 5 | 5 | 10 |
| 2 | 6 | 7 | 13 |
| 3 | 2 | 7 | 9 |
| 4 | 1 | 6 | 7 |
| 5 | 1 | 5 | 6 |
| 6 | 0 | 3 | 3 |
| Above 6 | 0 | 2 | 2 |
| | 15 | 35 | 50 |

*Previous attacks.* There are some diseases which, after having occurred once, confer on persons an immunity from subsequent attacks. This, however, is far from being the case with pneumonia, for of 78 children who came under my care for inflammation of the lungs, 31 were stated to have had previous attacks of the disease; 21 once, 4 twice, 2 four times, and 4 were said to have had it several times, though the exact number of seizures were not mentioned. Of these 31, 10 were under two years of age, 10 between two and three, and the remaining 11 between three and six. Except in a few instances, these statements are necessarily founded on the reports of the mothers of the children, or of other non-professional persons, and are therefore open to error; but at any rate they approximate to the truth, and may the more readily be trusted from their coincidence with the facts published by M. Grasolle, in his work on Pneumonia.

From the detail of the *post-mortem* appearances produced by pneumonia, and the investigation of the causes which give rise to its occurrence, we pass next to the examination of the symptoms by which it is characterised.

Symptoms of Pneumonia. Idiopathic pneumonia presents some differences in its course, according as it is or is not ushered in by catarrhal symptoms. The latter being the most usual may first claim our attention, after which any peculiarities may be pointed out which distinguish the form that supervenes on catarrh.

*First Stage.* Idiopathic pneumonia, unattended with catarrhal symptoms, is usually preceded for one or two days by a condition of general feverishness, exacerbated towards evening, with fretfulness, pain in the head, and great restlessness at night; or, if the child sleeps, its repose is unsound; it talks in its sleep, or wakes suddenly in a state of alarm. Cough comes on; at first short and hacking, but often it seems to cause no uneasiness to the child, and is so slight as hardly to attract the parent's notice. The thirst is considerable, the appetite impaired, the child showing distaste for solid food; or it begins to eat greedily, then suddenly leaves off, with the half masticated morsel in its mouth. The tongue and lips are of a florid red; the former is less moist than usual, and is generally coated in the middle with a thickish white fur. The bowels are generally constipated, and vomiting is not infrequent, especially in infants at the breast, who suck eagerly and by starts, then vomit the milk unchanged, and soon seek for the breast again. In them too, even in this precursory stage of pneumonia, the tongue is sometimes quite dry. If, while a healthy infant is sleeping, the mouth be gently opened, it will be observed that the tongue is applied to the roof of the mouth, and that respiration is carried on through the nares. So soon, however, as the lungs become affected, even when no other symptom exists than general febrile disturbance, and, perhaps, the vomiting above alluded to, the infant will be seen no longer to breath solely through his nose, but to lie with the mouth partly open, and drawing in air through it. This imparts to the tongue its preternatural dryness, and the same inability to respire comfortably through the nares causes the child to suck by starts. The infant seizes the breast eagerly, sucks for a few moments with greediness, then suddenly drops the nipple, and in many instances begins to cry. As the disease advances, these peculiarities in the mode of sucking and of respiration often become more striking, but it is at the onset of the disease that it is of especial importance to notice them, since they afford most valuable indications of its real nature. Their value too is so much the greater from their being independent of those accidental circumstances which may modify so many of the other signs of pneumonia. There is not always marked dyspnœa at this stage of the disease, and the frequency of the pulse and respiration is so much modified by position and other causes, that at the commencement of pneumonia very great dependence could not be placed on them, even if the fretfulness of the little patient, or its fears which the presence of the medical attendant often excites, permitted them to be counted with accuracy. Auscultation, too, if alone relied on, might possibly not guide the practitioner at once to the true nature of the disease; for all the above mentioned signs may exist, associated even with very notable increase in the frequency of the respiration, and in older children even with pain referred to the chest or abdomen, without any other auscultatory phenomena than intense puerility of the respiration with, perhaps, an occasional sibilus.

It is not always, however, that the advance of the first stage of pneumonia is so gradual as has just been described, for sometimes a child who has gone to bed well awakes towards morning in a state of alarm, refusing to be pacified, with a flushed face, and burning skin, and hurried breathing, and short cough. This sudden supervension of pneumonia is not so often met with among infants at the breast as among children from two to four years old. The alarming nature of the symptoms usually induces the parents to apply at once for medical aid, and cases which have commenced thus tumultuously appear to be as amenable to treatment as those which seemed at first far less violent. Even in cases where treatment has not been at once adopted, the storm generally subsides in twenty-four hours, and the disease passes into the second stage without presenting any further peculiarity.

*Second stage.* The first stage of pneumonia for the most part passes gradually into the second, the symptoms of disturbance of the respiratory organs becoming by degrees more and more apparent. Infants who hitherto had had moments of cheerfulness in the early parts of the day, or about noon, now no longer wish to be removed from the cradle or from the recumbent posture in their nurse's arm; and older children have quite lost all interest in their play, they become drowsy, ask to be put to bed, and cry if taken up. The respiration is now evidently hurried, the abdominal muscles are brought into play to assist in its performance, and the *alæ nasi* are dilated with each inspiration. The cough has become much more frequent; it still retains its hardness, but lasts longer, sometimes coming on in paroxysms, and often seems to cause pain, the child crying when it comes on, and laboring to suppress it, an effort which appears only to make it last longer and return more often. The bright flush of the face and the florid tint of the lips have gone, but the heat of skin continues. It is now a pungent heat, which becomes more sensible the longer the hand is kept in contact with the surface. It is often unequal, the trunk being intensely hot, while the extremities, particularly the feet, are cold; and on inquiry it will be found that the temperature varies much at different times. The face has assumed a puffed, heavy, but anxious appearance, and when the child is very young, or the pneumonia very extensive, the lips put on a livid hue, which is also very evident around the mouth, while the face generally is pale. Anorexia continues, but the first is generally very urgent, and, in children who are not at the breast, vomiting for the most part ceases. Infants who suck, still very frequently vomit the milk, perhaps owing to the urgent thirst they feel inducing them to suck too greedily, and thus overload their stomachs; since they generally vomit almost immediately after leaving the breast, while they do not reject small quantities of fluid given from a cup or spoon. It will also be observed that the respiration of a child is now greatly hurried by the effort of sucking, that he drops the nipple panting from his mouth, or has not breath sufficient to make the vacuum necessary to bring the flow of milk.

Auscultation now detects mucous or crepitant rhonchus at the lower part of each lung, in which situation the air enters less freely than elsewhere. There is much difference even in cases which appear closely to resemble each other as to the extent of lung through which crepitation is heard as well as in the character of the crepitation itself. Usually, however, the crepitation is confined to the infra-scapular region, and it is of that kind which is known by the name of *sub-crepitant.* The results of percussion of this stage are often not very marked, but frequently a diminished sonoreity of the lower parts of the chest, as compared with the upper, may be detected, and the impression conveyed to the finger is that of greater solidity below and above the scapula. This last sign is often very valuable, since it may be perceived at a time when the ear cannot clearly detect any actual dulness on percussion.

*Third stage.* In idiopathic pneumonia, unpreceded by catarrhal symptoms, death hardly ever takes place in this stage; but if the disease is left to itself, or if it is unchecked by the remedies employed, the second stage passes into the third; a transition which usually takes place in from twenty-four hours to three days. The respiration now becomes more labored, and though its frequency is sometimes diminished, it will be found to have become irregular; several short and hurried inspirations being followed by one or two deeper, and at longer intervals, and these again by hurried breathing. The cough sometimes ceases altogether, or if not, it is less frequent and looser, since it is now produced by the child's efforts to clear the larger air-tubes from the accumulating secretions. The voice is often lost, the patients speaking only in a hoarse whisper. The face looks sunken, the extremities are cold, and though the surface of the trunk is still hot, yet the skin has often lost something of its previous dryness, and clammy sweats break out, especially about the head. The pulse is extremely frequent and small, and its beats so run into each other, that it is almost impossible to count them. The child is extremely restless, tossing about from side to side, as much as its reduced powers will permit, or it lies in a state of half consciousness, though sensible when spoken to, and fretful at being disturbed. If raised hastily from the recumbent posture, or if put to the breast, the great increase of dyspnœa which is immediately produced, shows how seriously the respiratory organs are affected. In many cases too the livid hue of the face and of the nails are further proofs of the great impediments which exist to the decarbonization of the blood. This condition seldom lasts above two or three days; for either life becomes gradually extinct, without the supervention of any new symptoms, or convulsions occur which are followed by fatal coma, or the child recovers from it for a few hours, only to suffer a second attack of convulsions, and a return of the coma, in which state it dies.

The disease, however, does not always terminate fatally in this stage, but a kind of imperfect recovery sometimes takes place. A diminution is obvious in the more alarming symptoms; the patient begins to express some desire for food as well as drink, and even has occasional gleams of cheerfulness. The cough, which had almost or altogether ceased, returns, but it is hard and hacking as in the second stage, and though there is no urgent dyspnœa, the breath is habitually short. The skin is hot, dry, and harsh; the tongue is red, dry and sometimes chapped, or presents small aphthous ulcers at its edges; diarrhœa is not unfrequent; the child wastes daily, and dies in the course of a week or two, worn out and exceedingly emaciated.

The peculiarities which distinguish that form of pneumonia which is preceded by catarrhal symptoms, may be enumerated in a few words. The disease in this case often comes on insidiously, and developes itself gradually out of the preceding symptoms without its being possible to fix the exact date of its attack. At other times, however, there is a well-marked accession of fever and dyspnœa, and an aggravation of all the symptoms sufficient clearly to point out the time of the supervention of the pneumonia. The fever and heat of skin are less than in the other form of the disease, but the dyspnœa and distress are usually greater, and the face presents from the first a more livid hue. The cough is less hard, but it often comes on in paroxysms which greatly distress the patient; the respiration is more hurried and more irregular, and this irregularity comes on at an earlier stage of the disease. Mucous and sub-crepitant rhonchus are generally heard very extensively in both lungs, but the true pneumonic crepitation is unusual; and the inflammation being in these cases very often of the lobular kind, it may happen that no predominant affection of the lower lobes can be discovered. Head symptoms are more frequent; the patients' rest is disturbed, and they often mutter in their sleep, and have far more restlessness and jactitation when awake. Convulsions and coma more frequently precede death, and death occurs at an earlier period than in the other form of pneumonia.

Such is a sketch, confessedly a most imperfect one, of pneumonia in childhood. It may be well to attempt, by more minute details on some points, to fill up the numerous deficiencies in the portraiture. Sydenham indeed was able, within less space than this rude outline has already occupied, to draw pictures of diseases, so graphic and so true that all the observations of after years have found in them little to add, nothing to expunge. They, however, who can never hope to emulate so great a master, must content themselves with literally following his instructions, that "morborum phænomena clara ac naturalia, quantumvis minuta, per se accuratissime adnotentur; exquisitam pictorum industriam imitando, qui vel nævos et lævissimas maculas in imagine exprimunt."

The modifications in the character of the respiration in pneumonia, and the physical signs of the disease are of such importance as to merit most careful examination. Some acceleration of the respiration is almost always observed in cases of idiopathic pneumonia, but its degree affords no certain measure of the extent of the disease, nor will it be altogether safe to infer the absence of pneumonia, from the non-existence of dyspnœa. It is my belief, however, that marked dyspnœa will be found in all cases of lobular pneumonia, and also in all those cases of lobar pneumonia which have been preceded by catarrhal symptoms, or are associated with them.

It is often absent, or so slight as to be readily overlooked in the pneumonia which complicates diarrhœa, remittent fever, and other abdominal affections; as also in some cases where the thoracic symptoms are masked by the signs of cerebral or abdominal disease, constituting the *pneumonia larvata* of the old writers, in which, both for the aid afforded by auscultation, the true nature of the ailment would probably not be discovered. The frequency of the respiration does not in general continue progressively increasing from the outset of the disease to its fatal termination, but its maximum frequency usually coincides with that stage of the disease at which the crepitant or sub-crepitant rhonchus has attained its greatest extent, and sinks again when bronchial respiration and dulness on percussion indicate that the lung has become solid. When the attack of pneumonia is sudden and violent, and unpreceded by premonitory symptoms, the respiration sometimes reaches its greatest frequency within the first twenty-four hours; at a time when the auscultatory signs are scarcely developed, and when the ear detects nothing but intensely puerile respiration with occasional rhonchus or sibilus. In lobular pneumonia, which sometimes runs its course in four or five days, death taking place before any portion of the lung has become hepatized, the respiration may go on increasing in frequency until death. It is in such cases that I have observed the respiration more accelerated than under any other circumstances, and in one child so affected I counted 108 inspirations in the minute. Although the respiration often sinks in frequency on the supervention of hepatization it does not by any means return to a natural condition, but almost always becomes irregular. One or two slow inspirations are now succeeded by three or four very hurried, and it will be observed that now, while the help of the abdominal muscles is called largely into play, the lateral expansion of the chest is almost none. The marked influence too, of removing the child from the recumbent posture, and placing it in a sitting position, in causing great acceleration of the breathing, at once shows that the diminished frequency of the respiration is not the result of any favourable change. It appears then, that unquestionably valuable as are the indications furnished by the frequency of the respiration, there are many other points beside the mere number of inspirations which merit to be taken into account. Of greater value than the frequency of the respiration considered alone, is its frequency as compared with that of the pulse. Whenever with a diminution in the frequency of the respiration, the number of the pulse sinks too, we have a sign of amendment on which the most thorough reliance may be placed. It is of importance, however, always to bear in mind, how easily both the pulse and respiration are accelerated in young children; they ought both therefore to be counted while the child is in the recumbent posture, and before it has been disturbed or alarmed by auscultation, or by the examination of any other symptoms. It is better, too, if on any occasion this order of investigation cannot be followed, or if the child is fretful and cries at any attempt to ascertain these points, not to persevere, lest we be led by so doing to form erroneous conclusions as to the condition of the patient.

Physical Signs. The physical signs of pneumonia are no less important in the child than in the adult, but the practice of auscultation in the former is attended with difficulties which do not occur in the latter. To some, indeed, these difficulties have appeared so great as to induce them to regard the application of auscultation to young children as impracticable, whilst others insist on the insufficiency of the physical signs to establish an accurate diagnosis between bronchitis and pneumonia, and allude to cases in which "though unquestionable results of pneumonia were found after death, there was no crepitant rale and no bronchial respiration."* The valuable monograph of MM. Rilliet and Barthez, however, has sufficiently proved the possibility of deriving most important information from auscultation, and it is my conviction that with a little tact and a good deal of patience, pneumonia may be detected by its physical signs in the child, almost as surely as in the adult. It is true, that infants will not tolerate the application of the stethoscope, and that they will seldom allow any examination of the front of their chest; but the whole posterior part of the thorax may almost always be examined, and such an examination yields results which are of the greatest value. Iu practising auscultation, I have usually found it best to have the child taken from its cot and placed in a half-sitting posture in its nurse's arm. It is now possible, kneeling by the nurse's side, to listen to the whole posterior part of the chest unperceived by the infant, who does not appear to be incommoded by the pressure of the head against its back in the same manner as it is by the application of the stethoscope. Percussion too, may in this way be made upon the finger of the other hand so as to afford very useful information. Even in cases where children are most fretful and

* Maunsell and Evanson, op. cit. p. 306.

alarmed, and resist every attempt at auscultation, something may still be learned; for during the deep inspirations which interrupt their violent cries, air will enter freely, and from the sounds then heard by the attentive ear it will be easy to estimate the extent and stage of the disease.

It has seldom happened to me to see cases of infantile pneumonia from their commencement. In a few instances, however, when the onset of the disease has been sudden and violent, children have been brought to me within a few hours after the beginning of the attack. In these cases I have been able to confirm the observation of Dr. Stokes, that an intense puerility of respiration in the affected part will be found to be the principal phenomenon. On visiting these patients on the following day after their general symptoms have been greatly ameliorated by depletion and the employment of other remedies, I have no longer heard this intensely peurile respiration, but in its place the mucous or sub-crepitant rhonchus.

Cases such as those above described form the exception to the rule, and usually children did not come under my notice until the sounds indicative of increased secretion were audible in the lungs. These are either the mucous or sub-crepitant rhonchus or the true pneumonic small crepitation.

*Mucous rhonchus.* The mucous rhonchus is heard in most cases where catarrh has preceded the symptoms of pneumonia; it is, moreover, often heard in other cases of lobar pneumonia in the neighbourhood of the sub-crepitant rhonchus which usually occupies the lower and posterior part of the lungs. It is further heard occasionally in situations where the respiration has a distinctly bronchial character, and it very often persists in cases where convalescence has taken place, long after the disappearance of every other sign of pulmonary affection. It would be too much to assert that a portion of lung in which mucous rhonchus is heard may become solid without giving rise previously to any other physical sign, but it is certain, from the statements of MM. Rilliet and Barthez, that this change does sometimes occur so rapidly that bronchial respiration shall be heard to-day in a portion of lung, where on the day previous mucous rhonchus was the only sound the ear could detect. Of the accuracy of this statement I entertain no doubt, though I can confirm it from personal observation, only in as far as regards the *extension* of bronchial respiration not with reference to its *actual origin* in a lung. Often, however, I have detected bronchial respiration on one day occupying a very small portion only of one lung, while mucous rhonchus was heard in its neighbourhood, and on the following day much of this rhonchus has vanished and bronchial respiration has become well marked through twice or thrice its former extent. The occasional co-existence of mucous rhonchus with bronchial respiration is doubtless owing to the accumulation of secretions in the larger bronchi; hence, when heard under these circumstances it is an accidental phenomenon, and one of no value. The mucous rhonchus must be looked on as one of the least important of the physical signs of pneumonia, since it was present in thirteen only of fifty-one children under five years of age, in whom I carefully noted the symptoms furnished by auscultation. Nevertheless, greater value must, as MM. Rilliet and Barthez observe, be attached to it in the child than in the adult, since it is in the former, at least occasionally, the immediate precursor of bronchial respiration, while in the adult it is never the herald or any such grave occurrence.

*Sub-crepitant rhonchus.* The sub-crepitant is a sign of far greater importance than the mucous rhonchus, whether we regard the frequency of its occurrence or the consequences which follow it. It was heard in forty-two out of fifty-one cases; in thirty-one of which it either had not been preceded by mucous rhonchus, or if it had, that had ceased before the patients came under my notice. In thirteen cases it was associated with true crepitant rhonchus in some other part of the lung, or crepitant rhonchus succeeded it. In fourteen cases it was followed by bronchial respiration, and in six of these the bronchial respiration succeeded directly to it, without crepitant rhonchus having at any time been audible in those parts of the lung which became hepatised. Unlike the mucous rhonchus, it is not a transitory phenomenon continuing only for a few days, but it is a sign which, after the disease has once become established, persists until either the occurrence of mucous rhonchus in its place indicates that the lung is recovering, or the supervention of crepitant rhonchus or of bronchial respiration makes it evident that the morbid process is advancing unchecked.

*Crepitant rhonchus.* In twenty-two cases true crepitant rhonchus was heard, such as distinguishes the pneumonia of the adult. In fourteen cases it had been preceded by sub-crepitant rhonchus or was associated with it. In these instances it occupied a smaller extent of lung than the sub-crepitant rhonchus, and sometimes was confined to one lung while the sub-crepitant rhonchus only was heard in the other. Twice it succeeded directly to mucous rhonchus, and in six cases it was heard unattended either with mucous or subcrepitant rhonchus. In fourteen instances it was the immediate precursor of bronchial respiration, and was heard in parts of the lung near that in which the bronchial respiration was audible. The sub-crepitant rhonchus is sometimes a sign of the resolution of pneumonia, not so the crepitant, which I heard only when the disease was advancing, never at its decline, and its duration is far shorter than that of the sub-crepitant rhonchus, seldom exceeding two or three days.

It seems to be agreed on all hands that true pneumonic crepitation is of much less frequent occurrence in children under five years of age, than in the adult. Some writers indeed have gone so far as to deny its existence at this period of life, an opinion which MM. Rilliet and Barthez have shown to be erroneous. It does not, however, admit of doubt that the true crepitant rhonchus is decidedly less common in the child than in the adult, though it may not be easy to assign a satisfactory cause of this difference. Some value must probably be attached to the circumstance that while the frequency of the respiration in children is usually considerably increased under the influence of pneumonia, they no longer inspire so deeply as to fill the smaller air-vesicles, but the power of the respiratory muscles seems to be diminished, and the respiratory movements appear to be increased in frequency in order to compensate for their diminished energy. That some influence must be attributed to this cause is rendered further probable by the fact, that in the pneumonia of old persons, according to MM. Hourmann and Dechambre,* a similar absence of crepitant rhonchus is frequent. Another proof of this is afforded by the fact that in infantile pneumonia sub-crepitant rhonchus will frequently be the only sign perceptible until the child begins to cry, when in the deep inspiration which follows a fit of crying, the air that previously permeated only the larger air-tubes now enters the pulmonary vesicles, and at that moment distinct small pneumonic crepitation will be heard.

*Bronchial respiration.* Bronchial respiration was heard in 20 cases, in 5 of which it was detected in both lungs, in 7 it was heard only in the left lung, in 8 only in the right. It always existed in the infra-scapular region, though it was not by any means invariably confined to that situation. It sometimes supervened with great rapidity, occupying the whole of the lower half of one lung within twenty-four hours, and occasionally disappearing, as has been observed by Dr. Stokes

* Archives Générales de Médecine, 2e serie, tome xii. p. 45.

in the pneumonia of adults with similar rapidity, leaving no trace of its existence but large sub-crepitant rhonchus amounting almost to mucous rhonchus. Usually, however, it came on more gradually, occupying situations where crepitant or sub-crepitant rhonchus had been previously heard, and continued to be audible in cases which eventually terminated favourably for a week or even longer. Sometimes the bronchial respiration was unaccompanied by any other auscultatory sign in the same lung, but in the majority of cases sub-crepitant rhonchus was heard in its neighbourhood, and not unfrequently mucous rhonchus in its very situation. When resolution of the hepatised lung took place, I never heard a return of crepitant rhonchus, but sub-crepitant rhonchus in most cases became audible; in a few instances mucous rhonchus. In either case mucous rhonchus was eventually heard, and it often continued for many days after the lung had, in other respects, recovered its natural condition; apparently much as in the pneumonia of the adult, a prolonged expiration often persists for a long time after all the other signs of diseased action have disappeared. Bronchial respiration must be regarded as a very grave sign, since in 11 out of 20 cases in which it was heard, the disease had a fatal termination.

*Results of Percussion.* Though the results of percussion are decidedly less valuable than those of auscultation, yet this mode of investigation is by no means to be passed over as valueless. It is true that the great natural resonance of the chest in the young subject, the circumstance that both lungs are usually affected, and the restless fretfulness of the patient, render it less trustworthy than in the adult. Still a difference between the upper and lower part of the chest is generally appreciable long before bronchial respiration becomes audible; when bronchial respiration exists, dulness on percussion can always be detected, and even if it should be necessary to percuss with the utmost gentleness, so as scarcely to elicit a distinct sound, the finger is yet sensible of the presence of solid lung beneath. In cases, however, where the child cries even at the gentlest percussion, I think it better to give up the attempt, rather than by persevering in it to make the little patient dread the presence of his medical attendant, an occurrence which it is of extreme importance to avoid.

Diseases with which Pneumonia may be confounded. Before altogether dismissing the consideration of the symptoms of pneumonia, some notice must be taken of those errors of diagnosis into which the practitioner is most likely to fall. There are two stages of pneumonia, at each of which there is considerable danger of mistaking it for some other disease, namely, just at its commencement, and after it has existed for some considerable time.

*Hydrocephalus.* Pneumonia in its early stage may be mistaken for incipient hydrocephalus. The vomiting, pain in the head, restless nights with occasional wandering in the sleep, and the constipated state of the bowels common to both diseases, lead to this error. The cough in some cases of pneumonia is so slight as scarcely to be noticed; perhaps no catarrhal symptoms ushered in the disease, and not unfrequently the child's complaints are of his head, and of nothing else. But still there are circumstances, which, wholly independent of auscultation, would lead the careful observer to discriminate accurately between hydrocephalus, or cerebral congestion, and pneumonia. The vomiting in hydrocephalus is extremely frequent in its recurrence, the stomach immediately rejecting even the blandest fluid, and the matters vomited often have a greenish tinge, and this irritability of the stomach sometimes continues for days. The sickness in pneumonia resembles that which sometimes ushers in an attack of fever; it is violent, but does not in general continue long. The bowels are constipated in both diseases, but the evacuations of a hydrocephalic patient are either white from the complete absence of bile, or more frequently of a dark mud colour. The tongue in hydrocephalus is usually less furred, it is always of a less vivid red, the pulse, though frequent, has not the character of fulness, the heat of skin is far less, the thirst is absent. If these indications, however, be overlooked at the commencement of the attack, and if auscultation, by which the error might still be set right, be neglected, it is probable that each subsequent occurrence will be misinterpreted, and that the real nature of the disease will not be understood until it is revealed by the *post-mortem* examination. More or less sympathetic affection of the head is seldom wanting in pneumonia, to confirm the preconceived, erroneous notion; while as the child grows worse the difficulties in the way of making a careful auscultation increase. Convulsions too sometimes come on, and for days before the fatal termination the cerebral symptoms may be far more prominent than those indicative of affection of the lungs.

*Peritonitis.* Pneumonia may likewise be confounded with peritonitis or enteritis. The general febrile symptoms are common to both diseases, the little patient complains of pain in the belly, and cries, or show signs of uneasiness if pressure is made on the abdomen. The tongue is red, and in young children it often becomes dry from their lying with their mouth open; and poultices and leeches give at least temporary relief. It should, however, be borne in mind, that while pneumonia is a very common disease in childhood, acute peritonitis is one of very unusual occurrence. The child too does not select his posture with that great attention to guarding the abdomen, which he would display, if labouring under peritonitis. With reference to the complaint of pain in the abdomen, and its intolerance of pressure; it is of importance to remember that the statements of children with reference to the seat of pain are very vague, and that they frequently speak of pain in the belly when they mean the chest; while the impediment to the descent of the diaphragm occasioned by pressure on the abdomen, especially if this pressure is either sudden or considerable, will almost always excite expressions of uneasiness when the organs of respiration are in any way affected.

*Dentition.* Perhaps pneumonia is never so frequently overlooked as when it comes on in children during teething. It often happens among the poor that these cases are not brought to the medical practitioner until after the inflammation of the lungs has been proceeding for some days unchecked. Its early somptoms have probably been regarded merely as the catarrh which is incidental to children when cutting their teeth; and thus the parents, and sometimes too the medical attendant, allow the time for action to pass by unused. The disease in this case sometimes runs a chronic course, and its nature is further obscured by the tendency to diarrhœa, which exists during dentition, and which is now excited by the thoracic affection. This often becomes the most striking symptom, and all means are employed to suppress it, and to check the vomiting which generally attends it. These efforts, however, are unavailing, the child wastes daily, and its skin hangs in wrinkles about its attenuated limbs, while the abdomen becomes tumid, from the collection of flatus in the large intestines, and tender on pressure, and the tongue grows red, dry and chapped, or covered with aphthous ulcers. The cough now perhaps attracts notice, but its occurrence serves only to console the doctor with the belief that these symptoms have depended on phthisis, and that he has failed to afford relief because the disease was in itself irremediable. At last the child is worn out and dies, and great is the surprise to find no tubercle in any part of the body, no disease in the intestines; but pneumonia with purulent infiltration in both lungs; a disease which ought to have been detected, and which probably might have been cured.

Treatment. I will now endeavour to sum up as briefly as possible what I think I have learned of the treatment of this disease by careful and unprejudiced observation. I do so, however, with great diffidence, for I feel that none but they who have grown gray in the practice of their profession, and who can preface their remarks by appealing to "lengthened meditation, and to the diligent and faithful observation of many years," are in a position to claim for their opinions on such a subject any consideration. I can only plead my excuse, in the words of Sydenham; if indeed there be not something of presumption in borrowing the expressions of that great man: "Cæterum quantacunque fuerint aliorum conamina, semper existimavi mihi vitalis auræ usum frustra datum fore, nisi et ipse in hoc stadio versatus, symbolum aliquod, utcunque exiguum, in commune Medicinæ ærarium contribuerem."

*Depletion.* The first rank as a curative agent in the treatment of idiopathic pneumonia, I would unhesitatingly assign to depletion. It is true indeed that on this, as on some other points, my experience differs from that of French writers who have studied the disease at the children's hospital at Paris. One of these gentlemen* has affirmed that depletion, whether general or local, invariably debilitates the organism, and accelerates the fatal event. True as this assertion may be of pneumonia occurring under the peculiar circumstances which that institution presents, it cannot be extended to it as it is seen in this country. In no case of idiopathic pneumonia which came at an early stage under my notice, have I had occasion to regret the employment of depletion carried so far as sensibly to affect the system. In children of two years old and upwards I have usually resorted to venesection; but in younger subjects have contented myself with the application of leeches. From a child of two years old I have been accustomed to take four ounces of blood, and to direct the application of four or six leeches beneath the scapulæ if the symptoms should appear unrelieved after the lapse of a few hours. The effects produced by one bleeding in some of these cases have been most striking; the violent symptoms have sometimes yielded at once, and recovery has gone on uninterruptedly almost without the employment of any other

* M. Becquerel, in the Archives Générales de Médecine, 1839, p. 471.

remedy. Of the frequent repetition of bleeding in *these cases*, whether from the arm or by leeches, I have no experience, but my conviction is that children *generally* bear repeated bleedings ill, and I therefore do not practise them. That form of pneumonia which developes itself out of catarrh appears to be least under the control of depletion; no instance having come under my notice in which the attack was cut short by its employment, though here too, local bleeding at the outset is often beneficial.

*Tartar-emetic.* The tartar-emetic is a remedy of great value in the treatment of pneumonia; but I can by no means subscribe to the unqualified recommendation of it by some French physicians in all forms and at all stages of the disease. I would rather say of it what Wolfgang Wedel says of opium, "Sacra vitæ anchora, circumspecte agentibus, Cymba Charontis in manu imperiti." The cases in which it has seemed to me to be of the greatest service were those in which the pneumonia developed itself out of previous catarrhal symptoms, or in which it supervened on measles, or came on in the course of hooping-cough. In such cases, antimony given in doses of a quarter of a grain to a child of two years old, repeated every ten minutes, till free vomiting is produced, and afterwards continued every two or three hours for forty-eight or sixty hours, has often appeared to be of the most essential service, and the preservation of the patient's life has seemed in several instances to be due to its employment. In pneumonia, too, which has not been preceded by catarrhal symptoms; if after venesection the respiration still continues as hurried as before, and the condition of the patient has been apparently but little improved by that measure, tartar-emetic has seemed to be extremely useful. I have been accustomed to give it in large doses, as a quarter of a grain for a child two years old, and to repeat it every two hours for twenty-four hours, and have observed its use to be followed by a great diminution in the frequency of the respiration, and considerable relief to the distress of the patient; and believe that when given in these cases it paves the way for the advantageous employment of mercury. In no instance, however, in which pneumonia had been neglected, so that the period for depletion was past, and in which distinct bronchial respiration was audible, have I seen beneficial results from the employment of antimony in large doses, as recommended by many French practitioners. It is true that the heat of skin will subside and the respiration will diminish in frequency; thus inducing a treacherous appearance of improvement; but the strength of the patient is seriously impaired by it, the occurrence of a comatose condition, or of what the Germans have called the paralytic stage, will be hastened, and the fatal event accelerated, as I have learned by sad experience in cases where I gave this mode of treatment a full trial. It is not meant, however, that after the supervention of bronchial respiration, antimony ought never to be given, but only that under such circumstances it has appeared to me that it should not be employed except in small doses and in combination with other remedies.

*Calomel.* A very high rank as a remedial agent in the treatment of idiopathic pneumonia must be given to calomel. I have been accustomed, after due depletion, to administer calomel in doses of two grains, combined with a quarter of a grain of tartar-emetic, and half a grain of Dover's powder, and to repeat it every four hours for children of four years of age; diminishing the antimony after the lapse of twenty-four hours, if distressing sickness were occasioned by it, but persevering in the use of the calomel, provided the patient were not over-purged, until the disease began to yield or the gums showed signs of the mercurial action. The latter occurrence has been by no means frequent, and in no instance have I met with dangerous mercurial affection of the mouth from the employment of calomel in these cases. It has always been my custom to suspend the use of calomel for twelve hours immediately on the first appearance of mercurial affection, and if after the lapse of that time the signs of mercurialization had not increased, to return to its use in smaller doses, and repeated at longer intervals, provided the symptoms of pneumonia were so urgent as to demand its continuance. I have oftener been harassed by the purging which calomel induces, though this may be in a great degree controlled by combining it with Dover's powder. Besides the inconvenience and danger attending over-purging, calomel has seemed in some instances, especially in infants, to occasion a very harassing nausea and vomiting, which have rendered its discontinuance necessary, lest the patients should suffer from the want of sufficient nutriment. In such cases I have had recourse to mercurial inunction, and under its employment recovery has taken place even where circumstances had seemed to warrant none but a most unfavourable prognosis. It is especially in cases of neglected pneumonia, where the time for depletion has long gone by, where the administration of antimony is evidently contra-indicated by the exhausted state of the patient, and where the existence of diarrhœa forbids the employment of calomel, that the full value of this remedial agent is seen. I have employed it in the proportion of one drachm, rubbed into the thighs or axillæ every four hours, in children of four years of age. I have never observed salivation induced by it, but have seen the symptoms gradually diminish in severity during its employment, and the solid lung become once more permeable to air.

*Stimulants.* Usually, when the employment of mercurial inunction is indicated, the time for all directly depressing measures has long passed, and the best means of supporting the patient's strength become matter for serious consideration. No point in the treatment of the disease is more difficult than that of seizing the exact moment when the employment of stimulants becomes necessary, and no general rule for regulating their use can be laid down. It has, however, appeared to me to be seldom safe to withhold them when extensive bronchial respiration exists in a case on its first coming under the care of the practitioner, or when it has supervened in spite of active antiphlogistic treatment. If the patient, too, is beginning to be much purged; if the respiration is becoming more laboured and irregular, though diminished in frequency; aad if the pulse is becoming more frequent, and above all smaller and smaller, it is high time to resort to their use. Wine is as indispensable in such cases in the pneumonia of children as in that of the adult, and it may be necessary to give it even to infants at the breast. Ammonia may also be advantageously administered in this stage of the disease either in a mixture with the decoction of senega, or dissolved in milk which conceals its disagreeable pungency better than any other vehicle. If diarrhœa does not exist, strong beef-tea or veal-broth is the best form in which nutriment can be given; but if the bowels are relaxed, arrow-root or the *decoction blanche** of the French hospitals should be substituted for it.

*Blisters.* Blisters would probably be employed about this time in the treatment of pneumonia in the adult, but I cannot recommend their application in children. One objection to them arises from the time which elapses before they rise sufficiently, during the greater part of which they inflict considerable pain on the little patient, and make him extremely

* This, which is similar to the white decoction of Sydenham, is prepared by boiling half on ounce of hartshorn shavings, and the inside of a roll, in three pints of water, till reduced to a quart.

restless. The sores they produce when they cause vesication are sometimes very dangerous; they may even become gangrenous, and occasion the patient's death. Even if no such unfavourable results follow, they are nevertheless a source of constant annoyance to the child, at first from their soreness, afterwards from the troublesome itching which is felt when they begin to get well. Some pain is unavoidably produced whenever the dressings are changed, and the frequent repetition of this painful process sometimes makes the child, take a dislike to its attendants, who seem to it be the causes of so much suffering. It becomes suspicious of every one and quite unmanageable, while nothing is of greater moment than that a sick child should retain its fondness for its attendants during the whole period of its illness.

*Mustard-poultices.* It is quite possible that some of these objections may be obviated among the children of the wealthy by that care and those comforts which riches can always command, but I have been led, by the reasons above stated, to discontinue the employments of blisters in the treatment of pneumonia among the children of the poor. The same objections do not apply to the employment of mustard-poultices, and in many instances they have seemed to be productive of great good. They produce a much more speedy effect than blisters, and may be applied with safety over a much larher surface, hence they are often of signal benefit in relieving sudden accessions of dyspnœa; while from their not occasioning any breach of surface they may be reapplied as often as the emergencies of any case require.

Of the subsidiary medicinal agents, such as ipecacuanha and the class of expectorants, nothing need be said; but there are one or two points in the general management of the disease not altogether unworthy of notice.

*General management.* It is desirable, in all cases of pneumonia, at all severe, that infants should be taken from the breast, and that the mother's milk should for a time be given them from a spoon. This is of importance on two accounts; partly because the thirst they experience induces them to suck overmuch (hence it is well that barley-water or some other diluent be given to them frequently instead of the milk, in order that they may quench their thirst without overloading their stomach); partly, because the act of sucking is in itself mischievous, since, as must at once be perceived, it taxes the respiratory functions to the utmost.

A second important point is never to allow the children to lie flat in bed or in the nurse's arms, but to place them in a semi-recumbent posture in the arms, or propped up in bed. By so doing respiration is facilitated, since the diaphragm is relieved from the pressure of the abdominal viscera, and that stasis of the fluids in the posterior parts of the lungs is prevented, which has been shown by French writers to be so prejudicial to infants or children labouring under pneumonia.

The only other point to which I will allude is, that when pneumonia has reached an advanced stage, or has involved a considerable extent of the lungs, the children should be moved only with the greatest care and gentleness, lest convulsions should be brought on. Whatever may be the explanation of this occurrence, the danger is by no means an imaginary one, for I have seen instances in which children have been seized with convulsions immediately on being lifted somewhat hastily from bed and placed in a sitting posture; and on this account I have referred to what might seem to be a trivial matter.

*Bulletin of Med. Sciences.*

